# Hydrophilic Porous Polydimethysiloxane Sponge as a Novel 3D Matrix Mimicking Heterogeneous Pores in Soil for Plant Cultivation

**DOI:** 10.3390/polym12010140

**Published:** 2020-01-06

**Authors:** Feng Chen, Huihui Chai, Zhaoxi Song, Ling Yu, Can Fang

**Affiliations:** 1 Key Laboratory of Luminescent and Real-Time Analytical Chemistry (Southwest University), Ministry of Education, Institute for Clean Energy and Advanced Materials, Faculty of Materials and Energy, Southwest University, Chongqing 400715, China; cf19950629@email.swu.edu.cn (F.C.); chh0221@email.swu.edu.cn (H.C.); szx1227@email.swu.edu.cn (Z.S.); 2 School of Computer Science and Software Engineering, Southwest University, Chongqing 400715, China

**Keywords:** PDMS sponge, porous 3D matrix, artificial soil, seed germination, plant cultivation

## Abstract

In this work, a citric acid monohydrate (CAM)-templated polydimethylsiloxane (PDMS) sponge was proposed to mimic heterogeneous pore structures in the soil for plant cultivation. The porosity of the PDMS sponges was tuned by adjusting the CAM template. The water intake capability of the sponge was improved by (3-Aminopropyl) triethoxysilane (APTES) functionalization. The pore size and pore distribution were characterized by SEM and micro-computed tomography (micro-CT). The effect of pore structures on *Oryzasativa* (*O. sativa*) growth was investigated. Also, a 3D multi-layer PDMS sponge assembling was proposed to mimic the heterogeneous pore distribution at the different soil depth. The different growth rates of *O. sativa* and *Nicotiana tabacum L.* (*N. tabacum*) seeds on porous PDMS sponge indicated the impact of physical obstacles (pores) and chemical (water content) conditions on plant development. It is anticipated that this PDMS sponge could serve as a 3D matrix to mimic soil and provide a new idea for plant cultivation.

## 1. Introduction

Botanical research is a major field of life sciences, and progress in botanic science is closely related to economics and everyone’s daily life [1,2]. Because of its tremendous diversity in physical and chemical conditions, the soil itself is barely used as a standard culture based in a laboratory setting for plant cultivation. In a laboratory setting, to conduct plant cultivation, either a solid base, such as agar, or an aqueous solution is used as a culture base. For example, J. Malá et al. [3] used hydroponics as a culture base to study the impact of metal ions on plant growth. Agar is a jelly-like substance extracted from algal plants and is transparent at a certain concentration ratio [4]. The agar-based culture system is a platform widely used in plant root research [5]. Xu et al. [6] improved this method to allow roots and plant shoots to grow in dark conditions. Materials, such as hydroponics and agar culture, are still the most prominent, and the nonnegligible deficiency is that they do not truly reflect the structure of the soil. The physical structure of the soil is dominated by irregular particles and cavities, which are way more complicated than the water environment and agar [7]. Moreover, soil particle size fundamentally affects soil texture and soil moisture and, in turn, affect plant growth ultimately [8]. 

To better mimic and reflect the structure of the soil, materials, such as peat, vermiculite, perlite, slag, and quartz sand, have been used to support plant growth. The following conditions must be met for a good synthetic soil: no toxicity, able to supply water and oxygen to crops, and long-term stability [9]. For example, Lenzi A et al. [10] reported that the mixtures of fine/coarse perlite, peat/coarse perlite, and vermiculite/coarse perlite could mimic different soil structures. The impact of the composition of the mixtures on fertilization and growth of geranium was also investigated. Jacek D et al. [11] made soilless culture substrate with rye straw pieces, peat, and rock wool. Solid substrates such as sawdust [12], cotton fiber [13], glass ball [14], and silica gel [15], were also being used as artificial soil to support plant growth. However, there is a difficulty in characterizing the porosity/cavity of those materials, let alone finding a way to standardize them. 

Polydimethylsiloxane (PDMS) is a biocompatible synthetic material widely used in chemical, biological applications [16]. Most recently, seed germination [17] and root development of plant growth were investigated on PDMS-based chips [18,19]. Those studies prove that PDMS does not affect the healthy growth of plants. Inspired by the micro-replication method to generate micro-structured PDMS, this work proposed a citric acid monohydrate (CAM)-templet PDMS sponge as a 3D porous culture base to support plant growth. The micro-structures of PDMS sponge were characterized by scanning electron microscope (SEM) and micron computer tomography (micro-CT). The micro-structures of the PDMS sponge were tuned, and the relationship between plant root development and the porosity of PDMS sponges was studied. Also, a multi-layer PDMS sponge with distinct pore structures was proposed to mimic the soil with a different cavity at different depth. To the best of our knowledge, this is the first PDMS porous matrix applied in plant cultivation. It is anticipated this PDMS sponge will serve as a 3D matrix to mimic soil and provide a new idea for plant cultivation.

## 2. Materials and Methods 

### 2.1. Materials and Reagents

“Jinhui 10” rice seeds (*O. sativa*) were kindly gifted from Prof. Zhanglin Tang of Southwest University, China. *N. tabacum* seeds were purchased from Shangong (Shanghai, China). PDMS elastomer kits (Sylgard 184) were obtained from Dow Corning (Midland, MI, USA). Food color dyes (blue) were purchased from Dongguan Tianzhi Cai Food Factory, Dongguan, China. (3-Aminopropyl) triethoxysilane (APTES), CAM, and other chemicals were purchased from Aladdin Chemical Reagent Co, Ltd. Shanghai, China. Chemicals were used as received and prepared in ultrapure water (PURELAB flex system, ELGA Corporation, Paris, France) without further purification.

### 2.2. Preparation of Porous PDMS Sponge

**Scheme 1:**
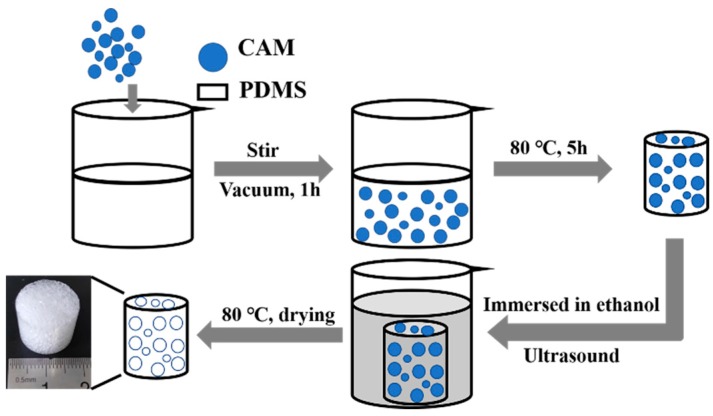
The procedures to prepare porous polydimethylsiloxane (PDMS) sponge.

Scheme 1 illustrates the procedure to fabricate a porous PDMS sponge. Firstly, the PDMS precursor: the curing agent was mixed thoroughly at a ratio of 10:1 (*w*/*w*). An indicated amount of CAM was added into the PDMS mixture and mixed thoroughly by stirring at least 10 min. Next, the PDMS and CAM mixture was carefully poured into a tube (diameter of 16 mm, height of 10 mm) and placed in a vacuum oven for 1 h to remove the bubbles within the mixture. Then, the PDMS-CAM mixture was cured in an oven at 80 °C for 5 h. The solidified PDMS-CAM block was removed from the mold and immersed in absolute ethanol to dissolve CAM under sonication. Then the sponges were washed by water for 5 times. Finally, the white PDMS sponge was dried in an oven at 80 °C for 5 h.

**Scheme 2:**
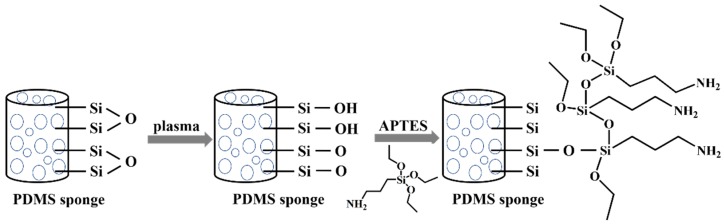
Surface modification of PDMS sponge. APTES: (3-Aminopropyl) triethoxysilane.

To improve the intaking water capability of PDMS sponge, APTES was used to modify the sponge (Scheme 2). In brief, the PDMS sponge was treated by oxygen plasma (Agilent Technologies Inc., Santa Clara, CA, USA) for 20 min and then immersed in 1% APTES/ethanol solution (*v*/*v*) for 24 h in the dark. After that, the PDMS sponge was washed five times with ethanol under sonication to remove excess APTES. Then the sponge was backed at 120 °C for two hours. In this way, the silanol group on the surface of the PDMS sponge and the silanol group of the APTES undergo a condensation reaction to form a siloxane bond (Si–O–Si) to coat APTES on PDMS sponge’s surface [20].

### 2.3. Characterization of the PDMS Sponge

The morphology of the PDMS sponge was examined by SEM (JSM-6510LV, JEOL, Tokyo, Japan). Before measurement, the non-conductive sponge was treated by tungsten sputtering for 120 s. The wettability of the sponge was characterized by using a dynamic contact angle measuring instrument (POWEREACH, Shanghai, China). Five spots were randomly tested for each sample. 

Porosity is an important parameter affecting fluid transport in porous materials. Here, to depict the pore distribution, the PDMS sponge was examined by using a micro-CT (CD-300BX/µCT, Chongqing Zhence Science and Technology Co., Ltd, Chongqing, China). In brief, sponges were placed on the stage, facing the scanning panel. The sample scan index was 1000 divisions, the exposure time was 1 second, and the scan time per sample was 17 min. The raw data was processed by ZCVAS, a software developed by Chongqing Zhence Science and Technology Co., Ltd (Chongqing, China). The raw data was imported into the ZCVAS software to reconstruct a 3D model of the samples. Moreover, section views at different axes can be extracted from the 3D model. The sectioned images were further analyzed by using the particle function of NIH Image J (National Institutes of Health, Image J 1.46, Bethesda, MD, USA). In brief, the section image was transformed from RGB format to 8-bit format, and the threshold was adjusted to separate holes and PDMS matrixes. The pore area was calculated using the “Analyze Particles” module of the NIH Image J software.

Next, the water displacement method was used to characterize the intaking water capability of PDMS sponges. First, the dry PDMS sponge was weighted as *M*_dry_, and the complete wetted PDMS sponge was weighted as *M*_wet_. The amount of water adsorbed by the PDMS sponge was calculated as:

Water content (W.C.) = *M*_wet_ − *M*_dry_

The void ratio (*e*) is a parameter that directly reflects the fluid transportation of a porous material. According to the literature [21], the *e* was measured by the liquid displacement method. In brief, water was used as the replacement liquid, dry PDMS sponge was immersed in distilled water, and the air within the sponge was pressed out. The *e* was calculated by the following formula:

*e* = *V*_w_/*V*_s_

where *V*_w_ is the maximum water content of PDMS sponge, and *V*_s_ is the volume of the PDMS sponge.

### 2.4. Plant Cultivation on PDMS Sponges

*Seed Preparation*: The *O. sativa* seeds and the *N. tabacum* seeds were soaked in deionized (DI) water at 37 °C for 48 h, with the DI water being changed every 12 h. The seeds were sterilized in 2% sodium hypochlorite for 3 min and washed five times with sterilized DI water. 

*Cultivation of Seed on Hydrophilic Porous PDMS Sponge*: Cylindrical PDMS sponges (16 mm in diameter and 10 mm in height) were placed in acrylic tubes (16 mm in diameter and 10 mm in height). The *O. sativa* seeds were planted on the sponge and incubated in an artificial climate incubator (LAC-250HPY-2, Shanghai Long Yue Instrument Inc., Shanghai, China). 200 μL DI water was refilled into the acrylic tube every 12 h. The growth status of the plants was monitored daily. 

*Cultivation of Seed on a 96-Well Plate Filled with Hydrophilic Porous PDMS Sponge*: The bottom of the 96-well microplate was removed. Cylindrical PDMS sponges prepared from a different CAM template (6 mm in diameter and 10 mm in height) were placed in each well. The *N. tabacum* seeds were planted on the sponge and incubated in an artificial climate incubator. 50 μL DI water was refilled into the micro-wells every 12 h. 

*Assembling of Multi-Layer PDMS Sponge to Mimic the Soil with Different Pores at Different Depth*: To mimic the soil with different porosity at different depth, a multi-layer heterogeneous PDMS sponge platform was assembled. In detail, three PDMS sponges prepared from PDMS-CAM ratios of 1:2, 1:3, and 1:4, respectively, were vertically stacked. The stacking was fastened in an acrylic tube (16 mm in diameter and 30 mm in height). The *O. sativa* seeds were planted on the sponge and incubated in an artificial climate incubator. 2 mL DI water was refilled into the micro-wells every 12 h. 

## 3. Results and Discussion

### 3.1. Improved Hydrophilicity to Ensure the Water Supplied for Plant Cultivation

**Figure 1:**
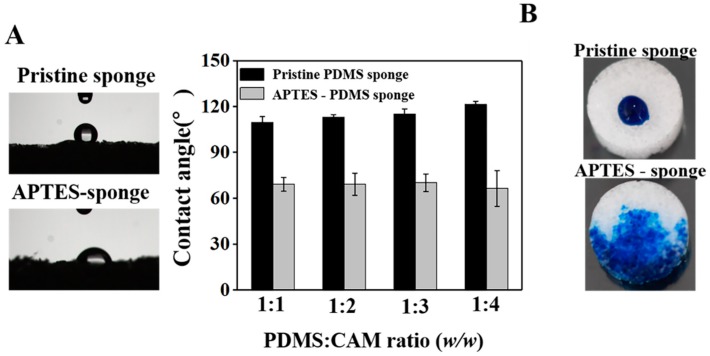
Wettability of the PDMS sponges. (**A**) Contact angle on the PDMS sponges before and after APTES modification and (**B**) water adsorbed by PDMS sponges. 100 µL diluted food color (**blue**) was cast on PDMS sponges. Pictures were taken 10 seconds after liquid casting. APTES: (3-Aminopropyl) triethoxysilane.

The wettability of porous PDMS sponge was characterized by contact angle measurement. As shown in Figure 1A, pristine PDMS has a contact angle (c.a.) of 108.92° ± 1.71°. Meanwhile, the c.a. of PDMS sponge prepared through the CAM template is 114.90° ± 3.44°. After APTES functionalization, the PDMS sponge has a c.a. of 68.84° ± 1.14°. Apart from contact angle characterization, the water permeability of the PDMS sponge was compared. Notably, 100 µL of diluted food color (blue) was cast on PDMS sponges. As shown in Figure 1B, a water drop forms on the pristine PDMS sponge. However, water quickly spread and penetrated the APTES-modified PDMS sponge. The impact of APTES modification on sponge water intaking were further studied. As illustrated in Supplementary Figure S1, the PDMS sponges were compressed and immersed into water. Then the force was released. After 1 min of immersion in water, the sponges were removed from the water. In this compress-release setting, the absorbed water-induced weight changes of pristine sponges and APTES-modified PDMS sponges were 122% and 124%, respectively, suggesting APTES-modification will not affect the mechanical force promoted water intaking. Next, dry pristine sponges and APTES-modified PDMS sponges were soaked in water for 1 min, and the absorbed water-induced weight changes were 1.6% and 23%, respectively, indicating more water can be actively absorbed by the APTES-modified PDMS sponge. APTES modification can improve the hydrophilicity of the sponges, which in turn improves the water-intaking capability of the PDMS sponges. Next, to evaluate if there is any residual CAM in the porous PDMS sponge, the prepared sponge was immersed in water and incubated at 25 °C for 3 days. The citric acid concentration was measured by the colorimetric method [22]. The result shows that the citric acid concentration is 0.23 ± 0.08 µg/mL. According to literature, citric acid is a chemical excreted by the root systems of some plants, and the detectable concentration is in the range of 201.5 µg/g root [23], 1863.5 µg/g root [24], and 576.36 µg/g root [25], which are 1000-times higher than the residual citric acid in PDMS sponge. In addition, to further avoid the interference of residual CAM in following plant cultivation experiments, all PDMS sponge were thoroughly washed with ethanol and water. 

### 3.2. Porous PDMS Sponge to Mimic Cavities in the Soil

**Figure 2:**
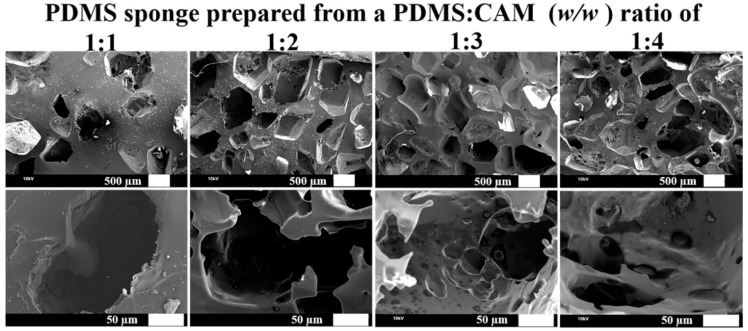
SEM characterization of the PDMS sponge.

First, the size of CAM particle was characterized by the sieve method [26]. Sieves with a grid number of 10, 20, 40, 60, 100, and 200 were used. The corresponding sieved particle sizes are 2, 0.9, 0.45, 0.3, 0.15, and 0.074 mm. As shown in Supplementary Information Figure S2, the CAM particles are mainly in the range of a few hundred micrometers to millimeters, suggesting heterogeneous pores would be obtained from CAM templating. Then, the morphology of the PDMS sponge was characterized by SEM. As shown in Figure 2, holes and cracks can be observed clearly. Apart from CAM particle size, with the increase of CAM amount in the PDMS-CAM mixture, the size of the holes/cracks increases. Connecting of pores leads to the merging of tiny cavities to form a larger one. The pore merging is most evident from the sponge prepared from a PDMS:CAM ratio of 1:4 (PDMS1:4). From SEM characterization, the size of the pores is in the range of 360 to 1075 µm. Less pore interaction was observed from PDMS sponge prepared from PDMS:CAM ratio of 1:2 and 1:3 (PDMS1:2, PDMS1:3). It is hard to find a pore connection from the sponge made from a PDMS:CAM ratio of 1:1 (PDMS1:1). SEM characterization indicates that the CAM template can form a cavity within the PDMS blocks. The size of the tiny hole can be tailored by adjusting the amount of CAM added to the PDMS precursor. 

**Figure 3:**
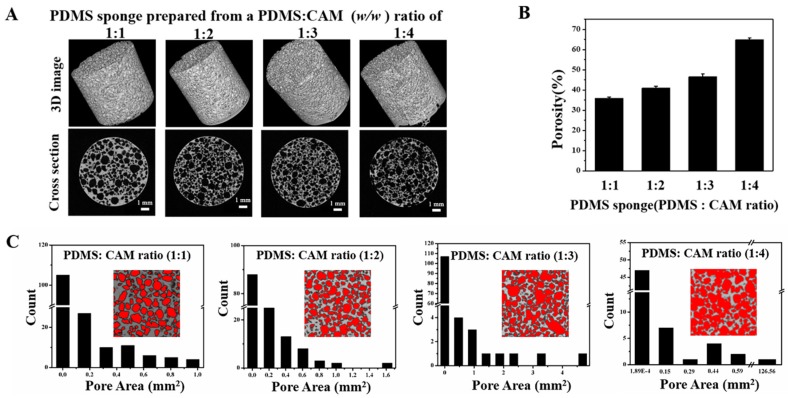
Micro-CT characterization of porous PDMS sponge. (**A**) Reconstructed 3D image of PDMS sponges and section view of PDMS sponges; (**B**) Pore percentages quantified by NIH Image J particle analysis; and (**C**) Pore distribution of PDMS sponges analyzed by NIH Image J particle analysis.

To further depict the pore size and pore distribution, micro-CT was applied for PDMS sponge characterization. Besides the 3D morphology, the horizontal and vertical section views of PDMS sponges randomly extracted from the 3D image were shown in Figure 3A. The dark part of the image is the pores, and the white part is PDMS. It can be seen intuitively from the sectioned picture that as the quality of the CAM in the PDMS-CAM increases, the pores increase, and the connectivity is greatly enhanced. Through the imaging process, the distribution, size, and percentage of the holes in the sponge can be quantified. As compared in Figure 3B, when increasing the CAM from 1:1 to 1:4, the average pore percentage increased from 35.82% to 64.88%. Figure 3C shows the pore distribution map of each PDMS sponge. As the amount of CAM increases, a large area of openings appears. Although the majority of the pores are over 0.005 mm^2^, the area of the largest hole has gradually increased from 0.9647 to 125.6 mm^2^, suggesting interconnections between pores. Next, soil specimens obtained from different places were analyzed by micro-CT. As shown in Supplementary Information Figure S3, soil samples have distinct pore sizes and pore distribution. Soil sample II has a relatively uniform pore distribution, while the pore size is smaller than that of sample I and III. More connecting pores can be observed in sample III. The micro-CT characterization of soil specimens proves that heterogeneous pore structures in the soil. By adjusting the amount of the CAM template, the overall porousness can be tailored nicely. In addition, CAM with a different size-range and more PDMS:CAM ratio could be tried out to obtain PDMS sponges with other porosity to better reflect the varied porosity of the soil. 

**Figure 4:**
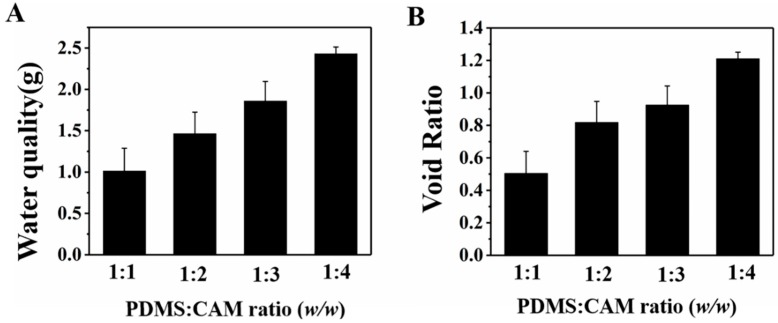
Water content of PDMS sponges. (**A**) The maximum water content of PDMS sponges. (**B**) Void ratio of PDMS sponges.

Next, the water content of PDMS sponges were characterized. As shown in Figure 4A, the maximum water content in PDMS sponges (16 mm in diameter, 15 mm in height, and 3014.4 mm^2^ in volume) fabricated from PDMS-CAM ratios of 1:1, 1:2, 1:3, and 1:4 are 1.01 ± 0.28, 1.46 ± 0.26, 1.86 ± 0.24, and 2.430 ± 0.08 g, respectively. The void ratio of different PDMS sponges showed in Figure 4B suggests that the effective porosity can be adjusted by the amount of CAM template used for sponge preparation. The higher amount of CAM resulted in a higher porosity, while dense PDMS sponges could be made by using less CAM template. Collectively, PDMS sponges could mimic different physical and chemical properties of the soil by controlling the water-conserving capability and the micro-structures, thus providing versatile platforms for plant growth.

### 3.3. Plant Growth on Hydrophilic Porous PDMS Sponge

**Figure 5:**
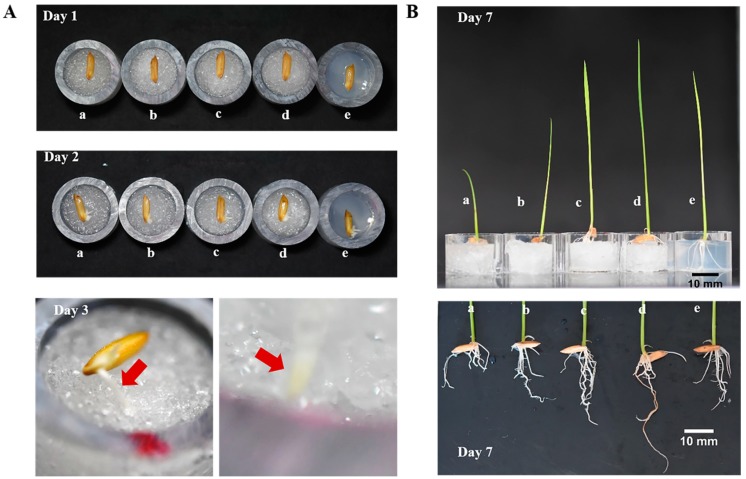
The growth of *O. sativa* on porous PDMS sponge. (**A**) Changes of seeds during the first 3 days of cultivation; (**B**) shoot and root development after 7 days of cultivation. (**a**–**d**) denote PDMS sponges prepared from a PDMS:CAM ratio of 1:1, 1:2, 1:3, and 1:4; (**e**) denotes 0.1% agar base.

The seed development on the porous PDMS sponges was recorded. As shown in Figure 5A, coleoptiles were observed sprouting out from the *O. sativa* seeds after 2-day incubation. On the third day, roots grew and extended into the porous PDMS sponge, which was prepared from a PDMS:CAM ratio of 1:4. *O. sativa* roots were observed penetrating PDMS sponge −1:3 and PDMS sponge −1:2 on day 4. It took five days for *O. sativa* seeds to grow into PDMS sponge −1:1. When the cultivation time was prolonged to 7 days, roots could be observed from the bottom side of the PDMS sponges −1:4, but not others. The seed germination in PDMS sponge-base is comparable to that in the standard agar base (Figure 5Ae). Apart from root development, the shoot length was compared. As shown in Figure 5B, with the increased porosity and water intake capability increase from PDMS sponge −1:1 to PDMS sponge −1:4, the length of the shoot also increases. The difference in root growth may be affected by the porosity of PDMS sponges. Comparing with the *O. sativa* grown in agar (Figure 5Be), the plants grown in PDMS sponges have different lengths of shoots, further suggesting the importance of tuning porosity in plant cultivation studies. As characterized in SEM and micro-CT measurement, less interconnected cavities exist in PDMS sponge prepared with less CAM template. The dense matrix imposes a stronger physical obstacle for roots to overcome and grow. Furthermore, less water intake in the denser PDMS sponge may also delay the development of plants. The results indicate that PDMS sponge could be a porous matrix, mimicking micro-cavity in the soil, while the water intake capability of hydrophilic 3D sponge provides water and nutrients for plant growth. Moreover, the porosity of the sponge can be tuned to mimic soil with different cavity/porosity. 

**Figure 6:**
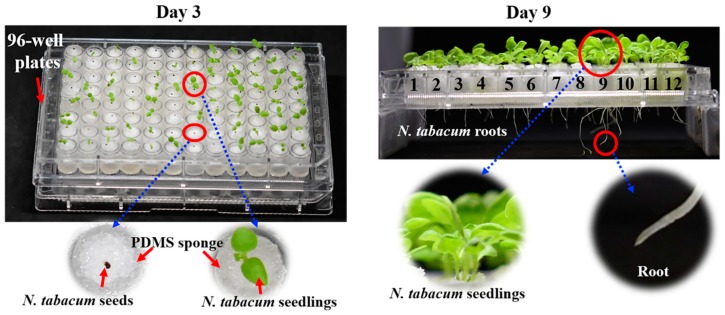
Growth of *N. tabacum* in 96-well microplate filled with PDMS sponges. Micro-well of columns 1–3, 4–6, 7–9, and 10–12 filled with PDMS sponges prepared from a PDMS: CAM ratio of 1:1, 1:2, 1:3, and 1:4, respectively.

High throughput is vital to seed quality evaluation. To this aim, the PDMS sponges were fabricated according to the size of the 96-well microplate, which is conventionally used in cell culture experiments. The micro-wells at columns 1–3, 4–6, 7–9, and 10–12 filled with PDMS sponges were prepared from a PDMS:CAM ratios of 1:1, 1:2, 1:3, and 1:4, respectively. A total of 96 *N. tabacum* seeds were placed in the micro-well, which was filled with Hoagland solution-wetted PDMS sponges. Figure 6 shows the seedling and root development after 3 and 9 days of cultivation. Shoots can be clearly observed from the microplate on day 3. Upon prolonging the cultivation time to 9 days, roots grew into the PDMS sponges and eventually penetrated through the sponges. The root structures can be easily compared because of the array format. The 96-well microplate format facilitates comparisons of plant growth at different culture bases, highlighting its potential in the high-throughput evaluation and screening of the seeds and plants. 

**Figure 7:**
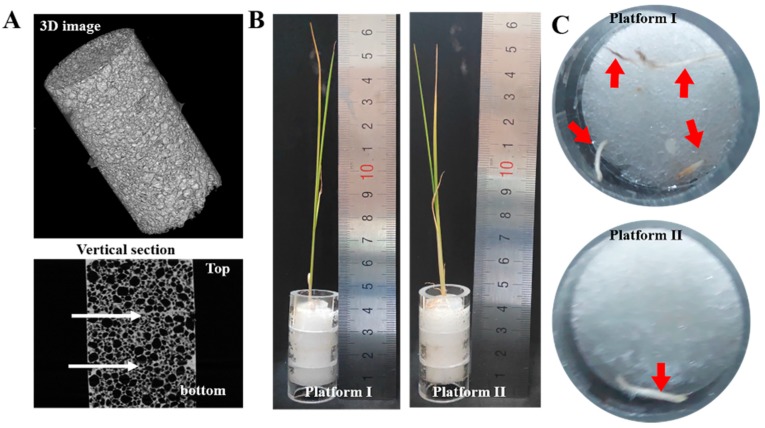
The multi-layer PDMS sponge lamination to mimic the heterogeneous soil. (**A**) micro-CT characterization of multi-layer PDMS sponge assembling, (**B**) growth of *O. sativa* seeds on multi-layer PDMS sponges, and (**C**) *O. sativa* roots development on multi-layer PDMS sponges. Platform I: the porosity from top to bottom is 40.89%, 46.56%, and 64.88%; platform II: the porosity from top to bottom is 64.88%, 46.56%, and 40.89%.

In real-world soil, the porosity may vary at different depth layers. Previous artificial soil and agar cannot mimic these variations. To this end, we proposed a layer-by-layer PDMS sponge model to establish a 3D matrix with different porosities at different depths. As illustrated in Fix, sponges prepared from different CAM templates were assembled vertically. As shown in Figure 7A, the top layer is the sponge with a porosity of 40.89%, while the middle and bottom layer of the sponge matrix have a porosity of 46.56% and 64.88%, respectively (platform I). The composition of another multi-layer PDMS sponge laminate (platform II) is opposite from the first one, as the top layer is the sponge with a porosity of 64.88%, while the middle and bottom layer of the sponge matrix have a porosity of 46.56% and 40.89%. Thus, the seeds growing on this 3D sponge will meet different geometry obstacles (porosity) and water supplies (water content). After 15 days of cultivation, the shoot and root from different multi-layer 3D PDMS sponge laminations were compared. As shown in Figure 7C, the *O. sativa* roots can grow through the first format multi-layer PDMS sponges, but not the other format. This top denser layer may delay the root growth into the matrix and limit water within the denser sponge, which will also retard the root growth. The heterogeneous microenvironment conditions would better reflect the real work situation than homogenous agar or water-based cultivation. The difference observed from two multi-layer PDMS sponge composites further suggesting the effect of matrix structures (porosity and pore distribution) and water intention on plant cultivation. Because of its excellent biocompatibility, tunable porosity, and water intake capability, the proposed 3-D porous PDMS sponge could be used as novel artificial soil for botanic studies.

## 4. Conclusions

A tunable porous PDMS sponge was proposed to mimic micro holes in the soil. A CAM template PDMS sponge was functionalized by APTES to improve the water intake capability and to provide sufficient water for plant growth. The 3-D pore structure of the hydrophilic PDMS sponge offers air and water for the growth of the plant therein. *O. sativa* seeds cultivated in a sponge with different porosity and water intake capabilities demonstrated different growth kinetics, indicating that a CAM-templated porous sponge can mimic physical microstructures of the soil and affect the plant growth. A 96-well high throughput platform and a multi-layer matrix with different porosity at different depth further demonstrated the potential of the PDMS sponge in mimicking the soil in plant research.

## Supplementary Materials

The following are available online at https://www.mdpi.com/2073-4360/12/1/140/s1, Figure S1: Compress-release induced water-intaking by PDMS sponge. A dry PDMS sponge is placed in a beaker. The sponge was compressed to its thinnest form. Water was added and immersed the compressed sponge. Release the sponge and keep it in water for 1 min. The passive absorption induced weight change was calculated as [(Mwet − Mdry)/Mdry] × 100%, where Mwet and Mdry are weight of water-intaking wet sponge and dry sponge, respectively; Figure S2: Particle size of citric acid monohydrate (CAM) characterized by sieving. 10 g CAM was analyzed by a sieve method [26]; Figure S3: micro-CT characterization of soil specimens.
