# Supplementary material for: Hydrophilic Porous Polydimethysiloxane Sponge as a Novel 3D Matrix Mimicking Heterogeneous Pores in Soil for Plant Cultivation
Source: Polymers (Basel). 2020 Jan 6;12(1):140. doi: 10.3390/polym12010140 (PMC7023002; doi:10.3390/polym12010140)
Supplement: Supplementary file 1 [file polymers-12-00140-s001.pdf]

polymers

Supplementary information

Hydrophilic porous polydimethysiloxane sponge as a novel 3D matrix mimicking heterogeneous pores in soil for plant cultivation

Feng Chen[1], Hui Hui Chai[1], Zhao Xi Song[1], Ling Yu[1*], and Can Fang[2*]

1 Key Laboratory of Luminescent and Real-Time Analytical Chemistry (Southwest University), Ministry of Education, Institute for Clean Energy and Advanced Materials, Faculty of Materials and Energy, Southwest University, Chongqing 400715, PR China; [2] School of Computer Science and Software Engineering, Southwest University,

* Correspondence: canfang@swu.edu.cn (C. F); lingyu12@swu.edu.cn (L.Y.) Tel.: +86-23-68254842

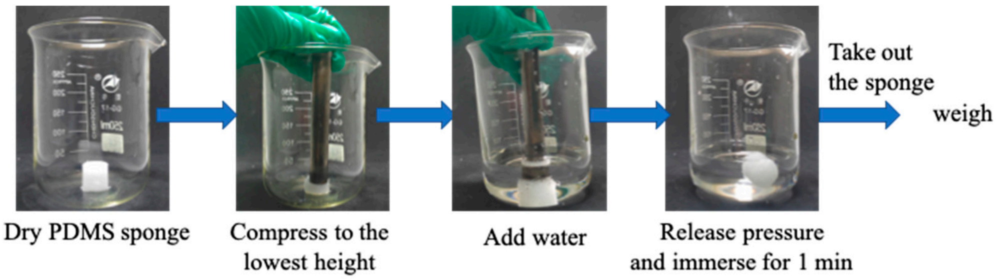

Figure S1. Compress-release induced water-intaking by PDMS sponge. A dry PDMS sponge is placed in a beaker. The sponge was compressed to its thinnest form. Water was added and immersed the compressed sponge. Release the sponge and keep it in water for 1 min. The passive absorption induced weight change was calculated as $[(M_{wet} – M_{dry})/M_{dry}]$ x 100%, where M_{wet} and M_{dry} are weight of water-intaking wet sponge and dry sponge, respectively.

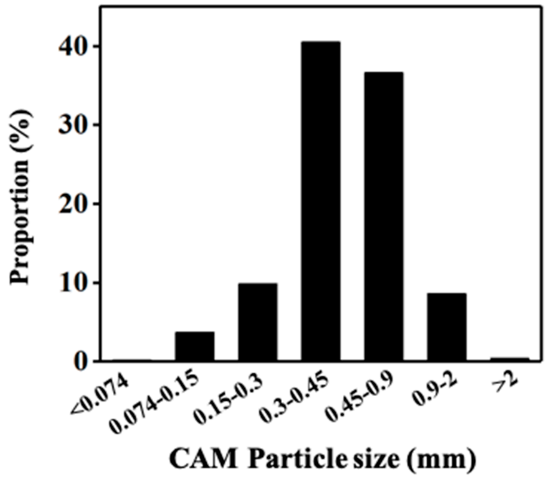

Figure S2. Particle size of citric acid monohydrate (CAM) characterized by sieving. 10 g CAM was analyzed by a sieve method [1].

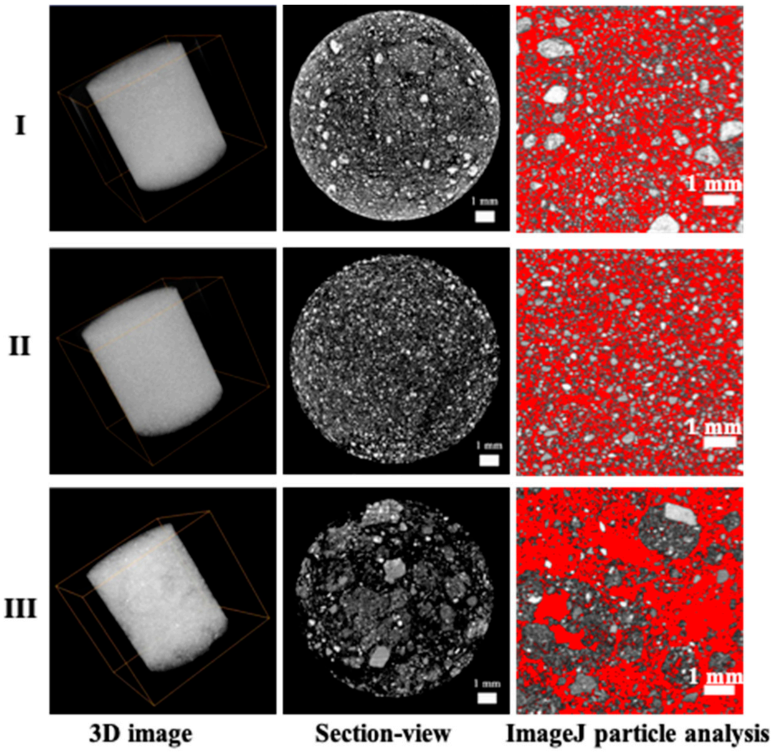

Figure S3. micro-CT characterization of soil specimens.

References

1. Dishman, K.; Doolin, P.; Hoffman, J. Comparison of particle size of cracking catalyst determined by laser linght scattering and dry sieve methods. *Ind Eng Chem Res*. **1993**, *32(7)*, 1457-1463.
